# Risk assessment of drug-associated miscarriage using XGBoost and SHAP explainability: a real-world pharmacovigilance analysis based on the FAERS database 

**DOI:** 10.3389/fphar.2026.1687391

**Published:** 2026-02-23

**Authors:** Sen Lin, Lanyue Ma, Ruiqi Zhao, Lisheng Peng, Xinyu Zhang, Bei Zhang, Danfei Li, Yijia Li, Li He

**Affiliations:** 1 Department of Oncology and Hematology, Shenzhen Traditional Chinese Medicine Hospital, Shenzhen, China; 2 The Fourth Clinical Medical College, Guangzhou University of Chinese Medicine, Shenzhen, China; 3 Department of Hepatology, Shenzhen Municipal Hospital of Traditional Chinese Medicine, Shenzhen, China

**Keywords:** disproportionality analysis, drug-associated miscarriage, FAERS database, immunomodulators, XGBoost

## Abstract

**Background:**

Miscarriage is a common and serious adverse pregnancy outcome. Assessing drug-associated miscarriage risk is essential for medication safety in pregnancy. Using the FDA Adverse Event Reporting System this study systematically mined adverse drug events related to miscarriage and combined machine learning with explainable Artificial Intelligence to evaluate potential high-risk drugs.

**Methods:**

We retrieved FAERS reports of miscarriage-associated ADEs from 2005 to 2024. Disproportionality analyses were conducted using the reporting odds ratio (ROR), proportional reporting ratio (PRR), Bayesian Confidence Propagation Neural Network (BCPNN), and Multi-Item Gamma Poisson Shrinker (MGPS), with subgroup analyses by age and body weight. An eXtreme Gradient Boosting (XGBoost) model was developed to predict miscarriage risk, and Shapley Additive exPlanations (SHAP) were used to interpret feature contributions. Finally, Weibull distribution modeling characterized the time-to-onset (TTO) from drug exposure to miscarriage.

**Results:**

A total of 36,389 ADEs were included. We identified several potential high-risk classes, notably immunomodulators, psychoactive/neuroactive agents, and antimicrobials. The XGBoost model showed favorable discrimination with a mean area under the curve (AUC) of 0.738. SHAP analysis reveals that immunomodulatory factors, such as adalimumab and infliximab, are significant predictors of miscarriage events in this model. The distribution of their SHAP values suggests a strong association between these drugs and miscarriage reports. time-to-onset analyses suggested that most miscarriages occurred within 2 years after drug exposure, with marked heterogeneity in risk timing across agents; anti-Tumor Necrosis Factor-alpha drugs (TNF-α) exhibited a higher early risk.

**Conclusion:**

Machine learning and SHAP interpretability analysis based on the FAERS database effectively identified immunomodulators, antiviral drugs, and psychiatric/neuropsychiatric medications as potential risk signals associated with miscarriage. These findings underscore the need for individualized medication assessment that considers patient age and body weight, providing evidence-based guidance and early alerting for reference for drug risk assessment during pregnancy.

## 1 Introduction

Miscarriage is among the most common adverse pregnancy outcomes, with a global incidence of approximately 10%–20%, the vast majority occurring within the first 12 weeks of gestation (Dimitriadis et al., 2020; Quenby et al., 2021). The World Health Organization estimated in 2023 that about 23 million miscarriages occur annually worldwide, leading not only to physical harm but also to psychological sequelae and socioeconomic burden. Epidemiological data indicate that miscarriage risk is influenced by multiple factors, including maternal age, prior miscarriage, chronic diseases, and environmental exposures (Magnus et al., 2019; Liu et al., 2025). For example, advanced maternal age (>35 years) is associated with a 2–3-fold increase in risk, and obesity (BMI > 30) has been identified as an independent risk factor (Lashen et al., 2004; Wang Q. et al., 2025). In low and middle-income countries, a higher proportion of miscarriages is attributable to infections, whereas in high-income countries, lifestyle and medication use play a larger role (Sethi et al., 2025; Jiang et al., 2025). These observations emphasize the importance of early risk assessment, particularly in the context of increasingly prevalent medication exposure.

Preconception and antenatal medication assessment is critical to safeguarding pregnancy. With the rising prevalence of chronic conditions such as diabetes, autoimmune diseases, and psychiatric disorders, medication use among women of reproductive age has increased substantially; approximately 50% of pregnant women use at least one prescription medication in early pregnancy, commonly analgesics, antibiotics, and antiemetics (Werler et al., 2023; Mansour et al., 2024). Preconception evaluation should account for potential reproductive toxicity, including disruptions to embryonic development and hormonal balance (Toledano et al., 2024). Clinical guidance from the American College of Obstetricians and Gynecologists, often based on limited evidence, stresses individualized assessment—for example, tailoring antibiotic regimens to age, body weight, and medical history (Acog Practice Bullet in, 2018). Nevertheless, real-world data suggest that many medication decisions during pregnancy lack systematic evaluation, leading to underrecognized risks (Lupattelli et al., 2014; Roldan Munoz et al., 2020; Lupattelli et al., 2020).

Drug-associated miscarriage refers to pregnancy loss directly or indirectly related to medication exposure during pregnancy or preconception. With the growing burden of chronic diseases and diversification of therapies, concerns about medication exposure in pregnancy have intensified (Chambers et al., 2021). The incidence of drug-associated miscarriage varies widely across populations and drug classes, reflecting not only inherent reproductive toxicity but also maternal comorbidities, genetic background, timing and dosage of exposure, and other factors (Bérard et al., 2019; Shehata and Nelson-Piercy, 2001). Several challenges complicate identification and prevention in clinical practice. First, ethical and safety constraints limit the inclusion of pregnant women in clinical trials, leaving most drugs with insufficient premarketing pregnancy safety data (Adam et al., 2011). Second, adverse drug events are underreported and often incomplete; outcomes such as miscarriage may be attributed to underlying disease or natural causes, delaying signal detection (Montastruc et al., 2011) Moreover, interindividual differences in drug metabolism, concomitant medications, and disease status further modulate risk, increasing the complexity of assessment (Huybrechts et al., 2020). Consequently, real-world pharmacovigilance databases such as FAERS play an increasingly important role in identifying drug-associated miscarriage risks, enabling large-scale, multicenter, multi-drug signal detection (Lavertu et al., 2021).

Current methods for assessing drug-associated miscarriage risk include case–control studies, cohort studies, and pharmacovigilance signal detection. Traditional disproportionality methods (e.g., ROR, PRR) are useful for signal screening but struggle to integrate complex clinical data or explain complex drug, disease, and population interactions. PRR compares the proportion of a specific adverse event (AE) among users of a particular drug to that among users of other drugs. ROR treats the database as a case–control study and requires excluding AE categories potentially linked to the drug under evaluation (Rothman et al., 2004). Disproportionality analysis quickly screens potential safety signals from large databases. However, it cannot control for confounding factors or capture complex nonlinear interactions (Wang B. et al., 2025). Therefore, ROR and PRR are difficult or impossible to distinguish differences in time, drug or event type reporting scores from differences in the incidence of adverse events. In contrast, XGBoost effectively models nonlinear relationships and complex interactions between features (Chen and Guestrin, 2016). SHAP then explains these model predictions, improving transparency. Integrating disproportionality analysis with machine learning and SHAP significantly enhances the reliability of safety signal detection (Chen and Lee, 2021). As artificial intelligence and machine learning continue to advance, multivariable risk prediction models are being increasingly utilized in drug safety research, thereby improving both the sensitivity and specificity of risk detection (Pilipiec et al., 2022; Lundberg et al., 2020). Moreover, model interpretability tools like SHAP provide novel approaches for clarifying mechanisms and facilitating individualized risk prediction (Dimitsaki et al., 2024; Lee and Chen, 2019).

Therefore, systematically integrating real-world data with machine learning and explainable AI holds promise for more rigorous and precise risk assessment and for informing clinical decision-making in the context of drug-associated miscarriage.

## 2 Materials and methods

### 2.1 Data sources and preprocessing

**FIGURE 1:**
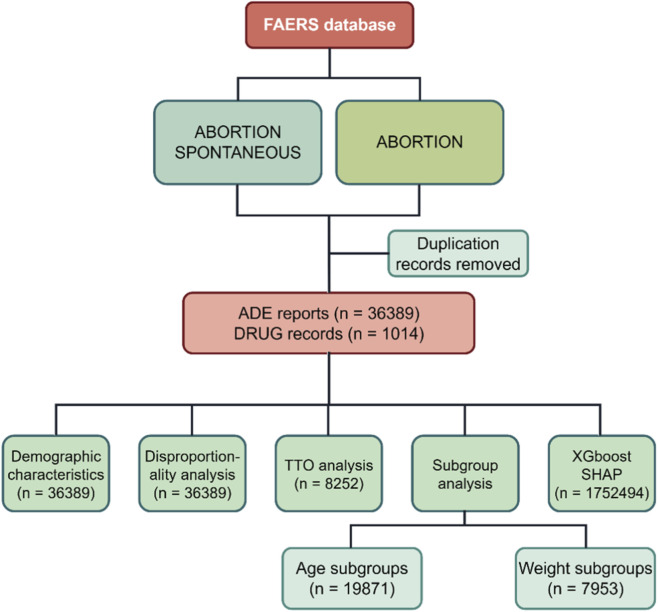
Schematic of the study design and analysis pipeline.

We extracted publicly available Individual Case Safety Reports (ICSRs) from FAERS spanning Q1 2005 through Q4 2024. Reports were submitted by healthcare professionals, consumers, and pharmaceutical manufacturers. ADEs were coded using Preferred Terms (PTs) from the Medical Dictionary for Regulatory Activities, version 27.0; (https://www.meddra.org/). At the PT level, we searched for “ABORTION SPONTANEOUS” and “ABORTION”. Report retrieval and deduplication followed FAERS guidance. This study strictly follows the case version deduplication guidelines recommended by the FDA for the FAERS database, utilizing core fields from the table to eliminate duplicate cases. In addition, we have implemented a step to exclude obsolete cases added after 2019, ensuring that only the most recent and valid case versions are retained. The specific rules and procedures are as follows: 1. Identify all related reports for each unique CASEID. 2. Retain the report version with the highest ISR number and/or the largest VERSION field value. 3. For records with the same CASEID and ISR, retain the record with the most recent FDA_DT (FDA receipt date). 4. When versions are identical, prioritize records with fewer missing data. These deduplication rules and procedures are based on the FDA’s official documentation: FDA Adverse Event Reporting System (FAERS) Quarterly Data Extract Files. This document is published quarterly alongside the FAERS ASCII/XML data compression package on the FDA website (https://fis.fda.gov/extensions/FPD-QDE-FAERS/FPD-QDE-FAERS.html). The document specifies that the FAERS database uses the case version as the core data unit, with CASEID serving as the unique identifier and FDA_DT as the report receipt timestamp. It further clarifies that case versions must be retained through field-level filtering, providing the foundation for the deduplication rules outlined above. To enhance attribution and reduce confounding, we included only drugs coded as primary suspect (PS) and excluded reports in which the drug role was secondary suspect (SS), concomitant (C), or interacting (I). We further restricted analyses to reports submitted by healthcare professionals and consumers. The detailed workflow for data identification, screening, and selection is shown in Figure 1. All statistical analyses and visualizations were performed in R (version 4.3.3). The R packages employed, along with their corresponding versions and functions, are as follows: XGBoost (v 1.7.11.1) was used to construct the gradient boosting decision tree model; data.table (v 1.17.8) and dplyr (v 1.1.4) were applied for large-scale data cleaning and refinement operations; caret (v 7.0–1) was utilized for data partitioning, hyperparameter tuning, and computation of model performance metrics; pROC (v 1.10.0.1) and PRROC (v 1.4) were employed to plot ROC and PR curves and to calculate the corresponding AUC values; parallel (v 4.5.1) was used to enable multi-core parallel processing for computational efficiency; ggplot2 (v 4.0.1) was applied for data visualization, patchwork (v 1.3.2) for combining multiple plots, and grid (v 4.5.1) for optimizing layout structures; shapviz (v 0.10.3) was used to compute SHAP values and conduct model interpretability analyses; openxlsx (v 4.2.8.1) was employed for exporting results in Excel format. All package versions correspond to the stable releases available during the study period, thereby ensuring the reproducibility and transparency of the analytical workflow.

### 2.2 Descriptive analyses

After identifying ADE reports related to drug-associated miscarriage, we summarized key characteristics, including demographics (age, weight), reporter occupation (OCCP_COD), reporting country (REPORTER_COUNTRY), and calendar year of report. These descriptive statistics characterize the study population and reporting patterns.

### 2.3 Disproportionality analyses

To detect ADE signals associated with drug-associated miscarriage, we conducted disproportionality analyses using two frequentist methods, ROR and PRR (Stricker and Tijssen, 1992; Evans et al., 2001); and two Bayesian methods, BCPNN and MGPS (Lindquist et al., 2000; Szarfman et al., 2002). Frequentist methods are advantageous for early signal detection (Montastruc et al., 2011; Rothman et al., 2004; Chen et al., 2008), whereas Bayesian methods tend to reduce false positives, provide narrower intervals, and are more sensitive when event counts are sparse (Matsushita et al., 2007). All four approaches were computed from 2 × 2 contingency tables. Calculation details and signal thresholds are summarized in Supplementary Table S1; exceeding a method’s predefined threshold was considered a signal of potential association between drug exposure and the ADE. After PT-level analyses, drugs meeting at least two methodological thresholds were retained for further correlation assessment to increase robustness. We interpreted the magnitude of each metric as a proxy for signal strength.

### 2.4 Subgroup analyses

FAERS provides sex, age, and weight. To explore heterogeneity in signals across populations, we performed subgroup disproportionality analyses stratified by age (<35 years vs. ≥35 years) and body weight (<70 kg vs. ≥70 kg). We visualized subgroup differences using volcano plots and forest plots based on RORs and corresponding p-values, comparing the distribution of potential miscarriage-inducing drugs between strata.

### 2.5 Machine learning model and SHAP explainability

Gain-based importance is an inherent metric within the XGBoost model. It is calculated by summing the information gain contributed by a feature across all decision tree node splits, thereby reflecting the feature’s contribution to the model’s accuracy. Specifically, it quantifies the extent to which the feature reduces the model’s prediction error. Essentially, this metric is focused on optimizing model performance. In contrast, SHAP-based importance is derived from the mean absolute SHAP values, which quantify the marginal contribution of each feature to the model’s prediction. SHAP aims to explain the impact of features on individual predictions by revealing both their overall importance and the direction of their association with the outcome. It identifies whether a feature contributes as a positive or a negative risk factor for miscarriage. The differences in rankings between these two metrics are mathematically justified: Gain-based importance tends to prioritize features with higher contributions to model splits and is more sensitive to data distribution and class imbalance. In contrast, SHAP-based importance, rooted in game theory principles, more effectively isolates feature interactions, making it more closely aligned with the clinical interpretation of “the actual impact of a drug on miscarriage risk.” Therefore, this study concludes that SHAP-based importance is more strongly associated with clinical miscarriage risk, and future interpretations of drug risk signals will primarily rely on SHAP importance rankings, with gain-based importance serving as an auxiliary reference during the model construction process.

We selected the 30 most frequently reported drugs (by ADE report count) for model development. Using ADE data for these drugs, we constructed a report-level feature matrix and binary outcome indicating whether the ICSR included a miscarriage event. We trained an XGBoost classifier to capture nonlinearities and higher-order interactions. Model performance was evaluated *via* 10-fold cross-validation, repeatedly splitting the data into training and test sets to mitigate overfitting and assess generalizability. Receiver operating characteristic (ROC) curves and AUC values were computed for each fold to summarize discrimination. To explain the fitted model, we applied SHAP, which quantify the contribution and direction of each feature to individual predictions and to the global model. SHAP analyses allowed us to rank drugs by their overall importance for miscarriage risk prediction and visualize how specific drugs influenced predicted risk. We integrated cross-validated performance metrics with SHAP results to appraise model stability, interpretability, and reliability. The Benjamini–Hochberg false discovery rate (FDR) procedure was applied to correct for multiple testing.

To strengthen the methodological foundation, this study draws on recent advances in pattern recognition and biomedical informatics, particularly the integration of deep learning and graph-based modeling for predicting complex biological relationships (Wei et al., 2026; Wei et al., 2025). This approach aims to better characterize the underlying structure of adverse event associations in the FAERS dataset, thereby enhancing the robustness and interpretability of the analysis results.

### 2.6 Indication confounding analyses

Indication confounding was defined based on the association between drug indications and spontaneous abortion risk factors (Xu et al., 2024). Specifically, drugs were annotated as “indication-confounded” if their approved clinical indications (per UMLS may_treat attribute) or target populations were strongly correlated with known spontaneous abortion risk factors, including: 1) autoimmune diseases (e.g., rheumatoid arthritis, Crohn’s disease), 2) reproductive tract infections, 3) metabolic disorders (e.g., diabetes, polycystic ovary syndrome), 4) thyroid dysfunction, and 5) progestogen use (targeting fertile-age women, the high-risk population for spontaneous abortion). This definition aligns with the core logic of indication confounding in adverse drug reaction studies: the observed drug-outcome association is driven by the underlying risk factor (indication) rather than the drug itself. This study utilizes may_treat for the analysis of indication confounding, aiming to eliminate the confounding framework between drugs and diseases.

### 2.7 Time-to-onset probability density analysis

To evaluate how risk evolves over time following exposure, we calculated TTO as the interval between drug start and ADE occurrence. Cases without reliable TTO information were excluded. We modeled TTO using the Weibull distribution, parameterized by the shape (α > 0) and scale (β > 0) parameters, which jointly determine the probability density and the temporal hazard pattern (see Supplementary Table S2 for formulas). In our context, α governs the hazard trajectory (decreasing, constant, or increasing) and β scales the distribution. We focused on α and its 95% confidence bound to classify risk dynamics: α < 1 and the 95% Confidence Interval (CI) < 1: decreasing hazard (early-failure pattern), α = 1: constant hazard (random-failure pattern), α > 1 and the 95% CI > 1: increasing hazard (wear-out pattern). We estimated Weibull parameters for all drugs combined and, separately, for adalimumab, certolizumab pegol, fingolimod, infliximab, interferon beta-1a, natalizumab, and vedolizumab to compare TTO patterns across drug classes implicated in miscarriage.

## 3 Results

### 3.1 Descriptive analysis

**FIGURE 2:**
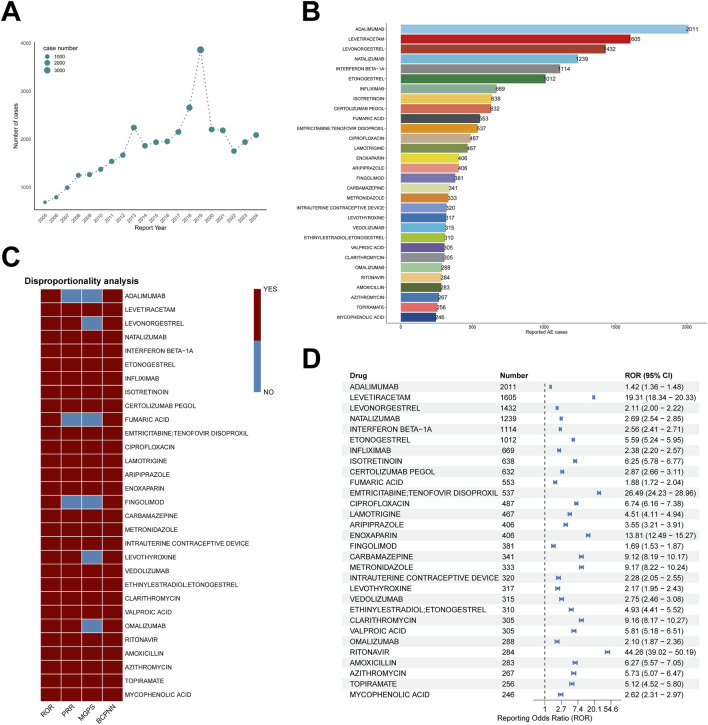
ADE signal detection results. **(A)** ADE reports from Q1 2005 to Q4 2024. **(B–D)** Summary for the top 30 drugs by reporting frequency, including **(B)** distribution of report counts for drug-associated spontaneous abortion, **(C)** threshold exceedance across the top 30 drugs for four disproportionality methods—ROR, PRR, BCPNN, MGPS and **(D)** ROR-based forest plot.

We analyzed FAERS data from Q1 2005 through Q4 2024; the case identification and screening workflow is shown in Figure 1. In total, 36,389 ADE reports involving 1,014 drugs were included. Annual report counts (Figure 2A) rose from fewer than 1,000 per year in 2005 to more than 2,000 per year by 2017. A sharp increase was observed in 2018–2019 (2,656 and 3,861 reports, respectively), followed by a return to approximately 2,000 per year during 2020–2024, comparable to 2013–2017 levels.

Demographic characteristics (Table 1) indicated, after excluding missing values, a predominance of females, middle-aged adults, and individuals with overweight/obesity. Among reports with age available, women of reproductive age accounted for 65.73% (*n* = 13,060) and those of advanced maternal age for 34.27% (*n* = 6,809). Among reports with weight available, 45.75% were <70 kg and 41.93% (*n* = 3,304) were ≥70 kg. By reporter type, 66.58% (*n* = 23,115) of cases were submitted by healthcare professionals (MD, HP, PH, OT). Geographically, reports predominantly originated from North America and Western Europe: 46.38% (*n* = 16,913) from the United States/Canada and 25.35% (*n* = 9,246) from major Western European countries (Germany, United Kingdom, France, Italy, the Netherlands, Sweden, Spain, *etc.*). Together, these patterns underscore that drug-associated miscarriage is a noteworthy concern and highlight the need for systematic risk assessment during medication use in pregnancy.

**TABLE 1:** Demographic characteristics of all ADE cases.

Characteristics	*N* = 36,389^a^
Age	32.0 [0, 84.0]
<35 years	13,060 (65.73%)
≥35 years	6,809 (34.27%)
Unknown	16,520
Weight	67.0 [0, 269]
<70 Kg	4,575 (58.07%)
≥70 Kg	3,304 (41.93%)
Unknown	28,440
Reported person	​
Health professional physician (MD)	11,207 (32.28%)
Non-healthcare professional consumer (CN)	11,372 (32.76%)
Pharmacist (PH)	774 (2.23%)
Health professional (HP)	3,080 (8.87%)
Other health professional (OT)	8,054 (23.20%)
Lawyer (LW)	228 (0.66%)
Unknown	1,674
Reported country	​
US	13,016 (35.69%)
CA	3,897 (10.69%)
UK	2,908 (7.97%)
DE	2,163 (5.93%)
FR	1794 (4.92%)
JP	740 (2.03%)
BR	712 (1.95%)
IT	688 (1.89%)
DK	460 (1.26%)
NL	448 (1.23%)
ES	413 (1.13%)
SE	372 (1.02%)
Others	7,056 (19.35%)
Unknown	1722

^a^ Median [Min, Max]; *n* (%).

### 3.2 ADE signal detection

At the PT level, we conducted disproportionality analyses and retained signals meeting the thresholds of at least two methods (including ROR). As shown in Figures 2B,C and Supplementary Table S3. We systematically excluded progestogens and related compound formulations. This prevented the original disease bias, where the drug’s use for miscarriage prevention could confound its association with disease progression. Specifically, the excluded drugs included: medroxyprogesterone, progesterone, intrauterine contraceptive device, ethinylestradiol/etonogestrel, etonogestrel, levonorgestrel, ethinylestradiol/norelgestromin, and ulipristal. After accounting for potential reporting bias affecting progestins (e.g., levonorgestrel, etonogestrel) in FAERS, four major drug categories emerged as potentially associated with miscarriage: immunomodulators (adalimumab, natalizumab, interferon beta-1a, infliximab, certolizumab pegol), psychiatric/neurologic agents (levetiracetam, lamotrigine, aripiprazole, valproic acid, carbamazepine), anticoagulants (enoxaparin), and antimicrobials (ciprofloxacin, metronidazole, clarithromycin, emtricitabine/tenofovir disoproxil, amoxicillin, azithromycin). Immunomodulators and psychiatric/neurologic drugs accounted for the largest numbers of ADE reports, notably anti-TNF-α or anti-integrin monoclonal antibodies used for autoimmune diseases—adalimumab (*n* = 2,011), natalizumab (*n* = 1,239), infliximab (*n* = 669)—and antiepileptics such as levetiracetam (*n* = 1,605), lamotrigine (*n* = 467), and carbamazepine (*n* = 341). Antiretrovirals also warrant attention: emtricitabine/tenofovir disoproxil (*n* = 537), tenofovir disoproxil (*n* = 202), and ritonavir (*n* = 284) showed substantial reporting volumes.

Forest plot results (Figure 2D; Supplementary Table S3) indicated that the above drugs were significantly associated with miscarriage reporting. Ritonavir showed an exceptionally strong signal (ROR = 44.26; 95% CI, 39.02–50.19), exceeding all other agents, followed by emtricitabine/tenofovir disoproxil (ROR = 26.49; 95% CI, 24.23–28.96) and levetiracetam (ROR = 19.31; 95% CI, 18.34–20.33). Enoxaparin (ROR = 13.81), clarithromycin (9.16), carbamazepine (9.12), metronidazole (9.17), isotretinoin (6.25), ciprofloxacin (6.74), amoxicillin (6.27), and valproic acid (5.81) all demonstrated strong signals (ROR > 5), warranting particular vigilance. Among immunomodulators, natalizumab (ROR = 2.69; 95% CI, 2.54–2.85), interferon beta-1a (2.56; 95% CI, 2.41–2.71), and infliximab (2.38; 95% CI, 2.20–2.57) exhibited moderate signal strength, while certolizumab pegol (ROR = 2.87; 95% CI, 2.66–3.11) and vedolizumab (ROR = 2.75; 95% CI, 2.46–3.08) showed slightly higher signals, suggesting the need for enhanced monitoring when these biologics are used in clinical practice.

### 3.3 Subgroup analyses

Using demographic variables (age and body weight), we performed subgroup disproportionality analyses. Note that these subgroup comparisons were conducted at the PT level and evaluate differences in reporting frequencies between strata; they do not establish causal relationships or adjusted associations.

**FIGURE 3:**
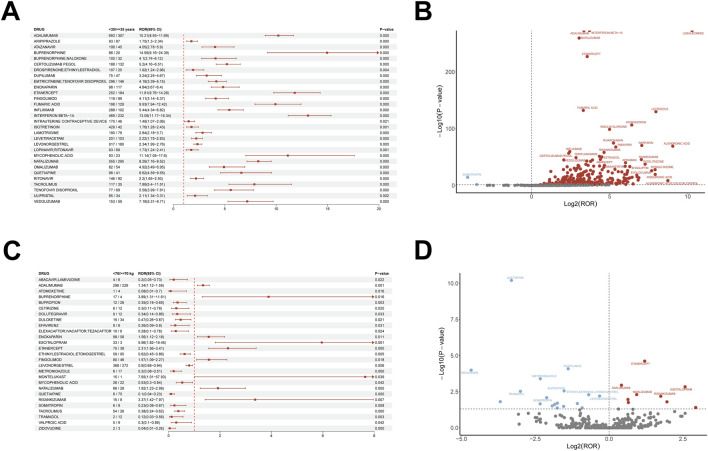
Subgroup analyses stratified by age and body weight. **(A, B)** Age-stratified ROR analyses with **(A)** forest plot and **(B)** volcano plot. **(C, D)** Weight-stratified ROR analyses with **(C)** forest plot and **(D)** volcano plot.

Age-stratified analysis (Figures 3A,B) showed that many drugs were associated with increased miscarriage reporting among women of reproductive age (<35 years), particularly immunomodulators. Adalimumab (ROR = 10.21; 95% CI, 8.93–11.69), natalizumab (ROR = 8.26; 95% CI, 7.16–9.52), and interferon beta-1a (ROR = 13.09; 95% CI, 11.17–15.34) exhibited strong signals, with adalimumab having the highest magnitude. Infliximab (ROR = 5.44; 95% CI, 4.34–6.82) and certolizumab pegol (ROR = 5.20; 95% CI, 4.16–6.51) showed moderate but notable signals. Beyond immunomodulators, ribavirin (ROR = 42.15; 95% CI, 21.41–83.00) and warfarin (ROR = 133.95; 95% CI, 39.84–450.42) displayed particularly strong signals, with warfarin markedly elevated.

Weight-stratified analysis (Figures 3C,D) indicated that among individuals weighing <70 kg, montelukast (ROR = 7.65; 95% CI, 1.01–57.93) and escitalopram (ROR = 5.96; 95% CI, 1.82–19.45) had the highest signals, suggesting heightened vigilance in lighter patients; buprenorphine (ROR = 3.89; 95% CI, 1.31–11.61) and risankizumab (ROR = 3.37; 95% CI, 1.42–7.97) also showed significant signals. In the ≥70 kg group, dolutegravir (ROR = 0.34; 95% CI, 0.14–0.86), valproic acid (ROR = 0.30; 95% CI, 0.10–0.89), and quetiapine (ROR = 0.10; 95% CI, 0.04–0.23) exhibited significant subgroup signals with ROR values below 1. Notably, immunomodulators such as adalimumab (ROR = 1.34; 95% CI, 1.12–1.59) and etanercept (ROR = 2.31; 95% CI, 1.56–3.41) retained positive signals in the <70 kg stratum. These findings suggest that patient body weight should be considered in prescribing decisions, with particular caution for montelukast and escitalopram in lighter patients.

### 3.4 Machine learning model and SHAP explainability

**FIGURE 4:**
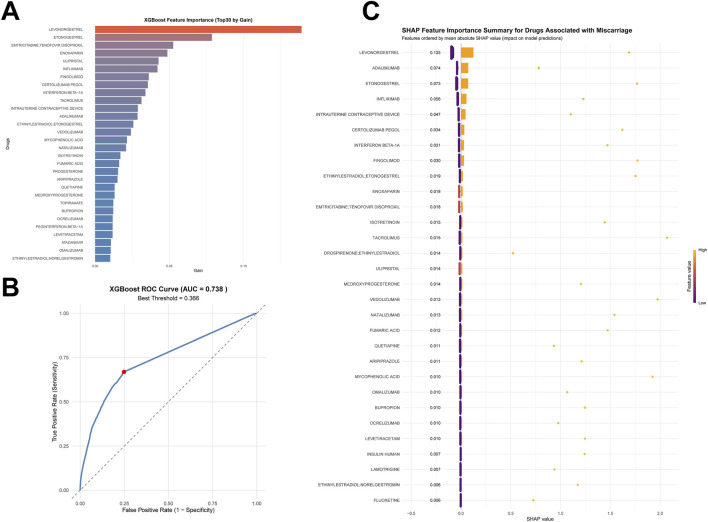
XGBoost modeling and SHAP explainability for the top 30 drugs to assess contributions to drug-associated spontaneous abortion. **(A, B)** Results of the XGBoost algorithm modeling, including feature importance values of the top 30 drugs **(A)**, and the ROC curve of XGBoost **(B, C)** SHAP interpretability analysis results of the XGBoost model, including a bar chart of overall feature importance values sorted by SHAP values and a hive plot.

Using the top 30 drugs by report frequency, we trained an XGBoost classifier to predict miscarriage risk (Figures 4A,B). The model achieved high overall accuracy on the test sets (accuracy = 0.8183; 95% CI, 0.8172, 0.8193). In 5-fold cross-validation (Supplementary Table S6), performance was consistent, with a mean AUC of 0.738 (Figure 4B).

The Benjamini–Hochberg FDR procedure showed that the results for the top 30 drugs were all statistically significant (Supplementary Table S4). Indicating reasonable discriminative ability. Feature importance analysis (Figure 4A) highlighted emtricitabine/tenofovir, enoxaparin, infliximab, fingolimod, certolizumab pegol, interferon beta-1a, adalimumab, and vedolizumab among the top contributors, many of which are immunomodulators. SHAP analyses (Figure 4C) further elucidated risk patterns: higher exposure to the top 30 drugs was generally positively associated with predicted miscarriage risk. Ranked by mean absolute SHAP values, the leading contributors were adalimumab, infliximab, interferon beta-1a, fingolimod, enoxaparin, emtricitabine/tenofovir disoproxil, and emtricitabine/tenofovir disoproxil. These results provide interpretable, model-based risk signals to inform medication safety during pregnancy.

### 3.5 Indication confounding analyses

Among the top 30 drugs ranked by Mean Absolute SHAP value, 15 drugs were identified as indication-confounded (Supplementary Table S7). The top two drugs (LEVONORGESTREL and ADALIMUMAB) were both confounded: LEVONORGESTREL is a progestogen used for contraception (targeting fertile-age women), and ADALIMUMAB treats autoimmune diseases (a well-established risk factor for spontaneous abortion). Notably, six of the top 10 drugs were confounded, highlighting the necessity of accounting for indication bias in interpreting drug-spontaneous abortion associations.

### 3.6 Time-to-onset analysis

**FIGURE 5:**
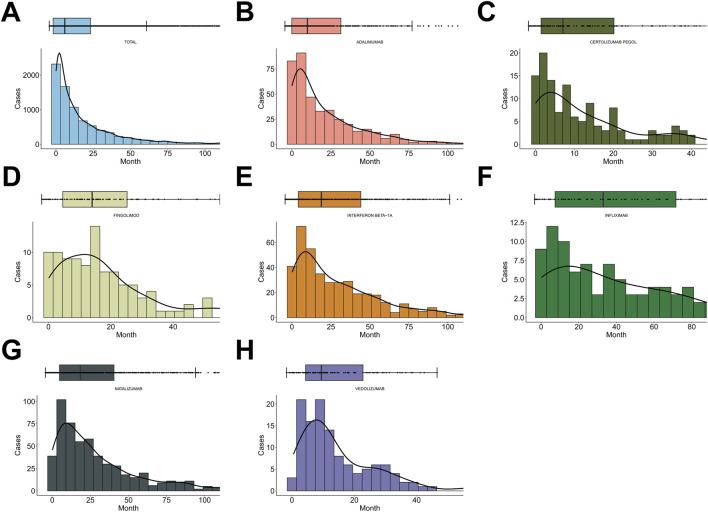
TTO analyses for immunomodulators. **(A)** TTO analysis for drug-associated spontaneous abortion across all drugs. **(B–H)** TTO analyses for key immunomodulators, including adalimumab **(B)**, certolizumab pegol **(C)**, fingolimod **(D)**, infliximab **(E)**, interferon beta-1a **(F)**, natalizumab **(G)**, and vedolizumab **(H)**.

To characterize how miscarriage risk varies over time following exposure, we calculated TTO and modeled its probability density with a Weibull distribution. A total of 8,252 cases had complete TTO data (Figure 5A; Table 2), approximately 70% of miscarriages occurred within 2 years of drug exposure, with a median TTO of 287 days. The Weibull parameters indicated an early-failure pattern: shape α = 0.73 (95% CI, 0.72–0.74) and scale β = 480.67 (95% CI, 465.69–495.66), consistent with a decreasing hazard over time.

**TABLE 2:** Parameters for calculating the probability density function of time-to-onset based on the Weibull distribution.

​								
Drug	Number	​			Weibull distribution			Failure type
		TTO (days)		Shape parameter		Scale parameter		
		Median (IQR)	Min-Max	α	95% CI	β	95% CI	
Total	8,252	287.00 (73.00–762.00)	1–11,396	0.73	0.72–0.74	480.67	465.69–495.66	Early failure
Adalimumab	417	397.00 (123.00–988.00)	1–5,235	0.82	0.76–0.89	648.67	568.98–728.35	Early failure
Certolizumab pegol	135	244.00 (89.50–604.50)	1–2,339	0.85	0.73–0.96	408.18	322.42–493.94	Early failure
Fingolimod	105	448.00 (189.00–756.00)	3–2,284	1.06	0.90–1.22	572.17	463.97–680.40	Random failure
Infliximab	97	1,007.00 (304.00–2070.00)	6–7,220	0.87	0.73–1.01	1,382.99	1,050.33–1715.63	Random failure
Interferon Beta-1A	410	643.50 (237.25–1,340.50)	5–6,004	1	0.92–1.07	922.83	828.50–1,017.15	Random failure
Natalizumab	549	634.00 (256.00–1,238.00)	2–5,264	1.04	0.97–1.11	916.52	838.85–994.20	Random failure
Vedolizumab	125	322.00 (182.00–689.00)	15–3,149	1.15	1.00–1.30	510.65	428.53–592.76	Degradation failure

We also examined TTO for key immunomodulators (Figures 5B–H; Table 2). Adalimumab (median TTO = 397 days; α = 0.82) and certolizumab pegol (median TTO = 244 days; α = 0.85) followed early-failure patterns. Fingolimod (median TTO = 448 days; α = 1.06), interferon beta-1a (median TTO = 643.5 days; α = 1.00), and natalizumab (median TTO = 634 days; α = 1.04) approximated a random-failure pattern (α ≈ 1), whereas infliximab (median TTO = 1,007 days; α = 0.87) suggested a slightly decreasing hazard with time and showed the longest median TTO. Vedolizumab (median TTO = 322 days; α = 1.15) exhibited a wear-out pattern, indicating increasing hazard with longer exposure. In terms of dispersion, certolizumab pegol had the shortest median TTO (244 days), while infliximab showed the broadest range (6–7,220 days). Consistent with the Weibull shape parameters, only vedolizumab (α > 1) demonstrated a clearly increasing temporal risk, whereas other agents displayed decreasing or approximately constant patterns.

These findings reveal pronounced temporal heterogeneity across immunomodulators: anti–TNF-α agents such as adalimumab and certolizumab pegol carry relatively higher early risks, whereas integrin inhibitors exhibit more complex time-dependent risk profiles.

## 4 Discussion

Using FAERS data from 2005 to 2024, this study systematically profiled the risk spectrum of drug-associated miscarriage by integrating multiple disproportionality methods, a machine learning model with SHAP explainability, and TTO analyses. Together, these approaches consistently highlighted several drug classes, including immunomodulators, psychiatric/neurologic agents, antimicrobials, and anticoagulants, as important contributors to miscarriage signals. The findings provide new evidence to inform medication safety in pregnancy and offer a foundation for individualized prescribing and refined pharmacovigilance strategies.

Our study identified a set of drugs associated with an elevated risk of miscarriage. These findings align with a growing body of real-world evidence. Proposed mechanisms for this association, as indicated by previous research, include immune dysregulation, oxidative stress, and vascular dysfunction (Agarwal et al., 2012; Guo et al., 2021; Yang et al., 2022). Consistent with prior literature, we identified immunomodulators and psychiatric/neurologic drugs as high-signal categories.

TNF-α supports implantation, placentation, and immune tolerance. Anti-TNF-α agents may disrupt the maternal-fetal interface by reducing inflammatory signals, potentially impairing embryonic development (Mor et al., 2011). Integrin inhibitors may also increase miscarriage risk by mediating autophagy and impairing trophoblast function (Wang R. et al., 2025). Clinical observations suggest that anti–TNF-α agents (e.g., adalimumab, infliximab) could interfere with implantation and placental development and raise the risk of early pregnancy loss (Dai et al., 2022; Garmendia et al., 2025). A meta-analysis reported significantly increased risks of preterm birth (OR = 2.62, P < 0.0001) and miscarriage (OR = 2.62, P < 0.0001) among pregnant women exposed to anti–TNF-α therapy compared with unexposed populations (Komaki et al., 2017).

Antiepileptic drugs can directly affect the placenta and fetus, increasing the risk of adverse pregnancy outcomes. They may disrupt fetal development by interfering with neurodevelopment, cell growth, and organ formation, raising the chance of miscarriage. Some Antiepileptic drugs also impair maternal nutrient metabolism, leading to deficiencies, or alter hormone levels such as thyroidhormones and progesterone, further affecting pregnancy. For example, valproic acid can harm the fetus through neurotoxicity, disrupt maternal hormones, and impair placental function, all of which heighten miscarriage risk (Ornoy et al., 2023). Antiepileptic drugs (AEDs) may cause oxidative stress, leading to fetal cell damage and early embryonic death (Ornoy, 2009). A multi-center study in Japan found miscarriage risk increases with higher AEDdoses and blood concentrations (Nakane et al., 1980). Levetiracetam and lamotrigine are linked to miscarriage, especially when used together early in pregnancy. Data from the Embryotox center showed dual therapy with these drugs significantly raised miscarriage risk (HR = 3.01) compared to unexposed pregnancies (Hoeltze et al., 2024). Aripiprazole and valproicacid are also associated with miscarriage, birth defects, and neurocognitive issues after prenatal exposure (Cho et al., 2025; Wang E. et al., 2025; Fietz et al., 2024; Stjerna et al., 2024). Although antibiotics are commonly used in pregnancy, certain agents (e.g., clarithromycin, metronidazole) have been linked to higher miscarriage risk and warrant cautious use (Muanda et al., 2017; Nguyen et al., 2025). These findings mainly come from pharmacovigilance and observational studies, as ethical concerns limit clinical trials in pregnant women.

Antimicrobial drugs, especially antiretroviral therapies, showed strong signals in our analysis. With the increasing use of antivirals in pregnant individuals with chronic viral infections (e.g., Human Immunodeficiency Virus, Hepatitis B Virus), the balance between preventing mother-to-child transmission and potential adverse pregnancy outcomes remains a critical concern. Multiple studies have associated antiretroviral exposure with increased risks of miscarriage, preterm birth, and low birth weight (Newell and Bunders, 2013; Zash et al., 2019). However, real-world studies present conflicting findings regarding the relationship between these drugs and miscarriage. For example, the Women’s Interagency HIV Study (WIHS), a multi-center study, found that women receiving Antiretroviral Therapy (ART) had a lower risk of miscarriage compared to those not receiving ART (Wall et al., 2019). In contrast, data from the Promoting Maternal and Infant Safety (PROMISE) trial indicated that individuals on ART, particularly those receiving protease inhibitors, had a higher likelihood of miscarriage or stillbirth compared to those who initiated ART during pregnancy (Hoffman et al., 2019; Theron et al., 2021). The potential biological mechanisms linking antiretroviral drugs to miscarriage include: First, the initiation of ART prior to pregnancy, which may increase the risk of maternal blood vessel insufficiency in the placenta, leading to placental dysfunction and miscarriage (Ikumi et al., 2021). Second, the exacerbation of oxidative stress by antiviral drugs, which may adversely affect fetal development and increase the risk of miscarriage (Costa et al., 2024). For pregnant women with HIV, the benefits of using antiretroviral drugs are clear, but it is crucial to balance the prevention of mother-to-child transmission with the risks of adverse pregnancy outcomes such as miscarriage, preterm birth, and low birth weight.

The TTO analyses add a temporal dimension to these findings. Most miscarriages occurred within 2 years of exposure, and certain immunomodulators (e.g., adalimumab, certolizumab pegol) exhibited early-failure patterns, whereas others, such as infliximab, showed broader and more complex temporal profiles. The Weibull modeling further revealed heterogeneity in hazard trajectories across agents, with vedolizumab demonstrating an increasing hazard over time. These results underscore the importance of exposure timing and duration when assessing risk in women who are pregnant or may become pregnant, and they support dynamic monitoring and periodic risk reassessment during therapy.

Although time-to-event analysis indicates that most miscarriage reports are concentrated in a relatively short period following drug exposure, this result is constrained by the time-recording limitations of the FAERS database and should not be directly interpreted as the temporal relationship between drug exposure during pregnancy and miscarriage. Specifically, for chronic disease medications like infliximab, the observed ‘long exposure time’ more likely reflects the patient’s long-term treatment history rather than the actual duration of drug use during pregnancy. Therefore, the TTO analysis results in this study should be viewed as exploratory findings aimed at generating hypotheses. These findings need further validation through specifically designed pregnancy cohort studies that can accurately record pregnancy start and end dates, pre-conception medication history, and detailed drug use during pregnancy. Additionally, we emphasize that future studies on pregnancy-related adverse events using spontaneous reporting databases should incorporate clinical expertise to properly filter time variables, minimizing the impact of data biases.

Using data from the FAERS database, this study systematically identified several drug classes associated with increased miscarriage risk, notably immunomodulators, psychiatric/neurologic agents, antimicrobials, and anticoagulants. We further characterized the temporal dynamics and interindividual heterogeneity underlying these drug-associated risks. The combination of machine learning and explainability offers complementary tools for pharmacovigilance and risk stratification. Future research should prioritize multi-dimensional data integration and mechanistic studies to enable more precise and intelligent medication safety management in pregnancy.

A key strength of this study is the rigorous accounting of indication confounding tailored to spontaneous abortion, a critical validity threat in pharmacoepidemiological research on adverse pregnancy outcomes. Unlike previous studies using generic may_treat annotations, we focused on risk factors specific to spontaneous abortion (e.g., autoimmune diseases, reproductive tract infections, metabolic disorders), ensuring accurate identification of confounded signals. The high proportion of confounded drugs in the top-ranked list (15/30) underscores the complexity of interpreting drug-pregnancy outcome associations: many drugs are prescribed to women with underlying conditions that inherently increase abortion risk, making it essential to distinguish between true drug effects and indication-driven bias. Our annotated list provides a valuable resource for clinicians and researchers to prioritize drugs for further causal validation (e.g., *via* propensity score matching or randomized controlled trials) while avoiding false positives from indication confounding.

This study has several limitations, primarily stemming from the inherent characteristics of pharmacovigilance databases:

First, the FAERS database relies on spontaneous reporting, which is susceptible to reporting bias, incomplete data, and challenges in establishing causality. Certain medications (e.g., progestins) may have their risks either overestimated or underestimated due to factors such as indications or reporting patterns. Moreover, FAERS data can only establish correlations, not causality. The scarcity of reports on drug-associated miscarriages, the inherent noise in spontaneous reporting systems, and indication confounding limit the potential for improving model precision. As a result, this study serves as an exploratory signal detection for adverse drug reactions rather than a confirmatory risk assessment. The drugs identified in this study should be regarded as potential warning signals for miscarriage. These signals are preliminary and necessitate further validation through well-designed case–control or prospective cohort studies.

Second, miscarriage is influenced by various confounding factors. Underlying conditions being treated with certain medications (e.g., immunomodulators, antivirals, psychotropic or neurological drugs), such as HIV or epilepsy, may independently elevate the risk of miscarriage. Additionally, concomitant drug use complicates the ability to isolate individual variables. Lifestyle factors or unrecorded accidents may introduce additional confounders that cannot be fully controlled. To mitigate these issues, future studies should integrate multiple databases for cross-validation, reducing bias introduced by relying on a single data source.

Third, The drug-miscarriage association signals identified through four disproportionate analysis methods and the XGBoost/SHAP model in this study are applicable only to clinical scenarios similar to the study population’s characteristics—primarily pregnant individuals in specialist medical settings in the US and Western Europe. These findings should not be directly extrapolated to clinical drug treatment decisions for pregnancy in other global regions.

Finally, the results of the subgroup analysis have certain limitations: As a spontaneous reporting database, FAERS relies on voluntary reports and lacks mandatory standardized data collection requirements. Among the demographic variables, age and weight exhibit high rates of missingness and non-random missingness; in clinical practice, information such as weight and age is often omitted as it is not considered core medical indicators. Moreover, the missing data is related to the severity of the patient’s condition and the degree of concern from the reporters, leading to non-random missingness. A prospective registered study found that the miscarriage risk is lowest (10%) for women aged 25–29, rises sharply after age 30, and is 53% for women aged 45 and older, which generally leads to a larger number of pregnancies among women under 30, thus resulting in more reported drug-associated miscarriages (Magnus et al., 2019); therefore, when calculating ROR stratified by age, the risk estimate for the <30 years subgroup may be biased, leading to distorted results that either overestimate or underestimate the actual risk for that subgroup. This may weaken or reverse the existing risk stratification pattern. For drugs with smaller sample sizes (for example, <50 reports), subgroup analyses are limited in stability and reliability due to insufficient statistical power. Therefore, in this study, we have explicitly defined the scope of subgroup analyses to only include drugs with report counts ≥100, and we reported the sample size for each drug (e.g., Infliximab, *n* = 97; Fingolimod, *n* = 105). For rare drugs, we only present the overall association signals without conducting further subgroup stratification. Although this study utilized a machine learning model to extract associations from the overall data, the aforementioned fundamental limitations mean that the results of the stratified imbalanced analysis based on incomplete demographic variables should be regarded as exploratory findings, and their interpretation should be approached with caution, pending further validation in prospective studies with complete baseline data or well-designed observational studies.

While machine learning models have enhanced predictive capabilities, their findings require further validation using prospective clinical data and an understanding of the biological mechanisms and individual susceptibility to drug-associated miscarriages. Additionally, promoting the standardization and data sharing of pharmacovigilance databases will improve the accuracy and timeliness of signal detection. Clinically, greater attention should be paid to risk assessment and personalized management of medication use during pregnancy, especially in monitoring high-risk drugs and developing alternative therapeutic options.

## 5 Conclusion

Leveraging FAERS data with machine learning and SHAP explainability, we identified immunomodulators, antiviral agents, and psychiatric/neurologic drugs as core risk contributors to drug-associated miscarriage. These findings support individualized prescribing that accounts for patient age and body weight and provide early risk alerts to enhance medication safety during pregnancy.

## Data Availability

The original contributions presented in the study are included in the article/Supplementary Material, further inquiries can be directed to the corresponding author.

## References

[B1] Acog Practice Bulletin (2018). No. 199: use of prophylactic antibiotics in labor and delivery. Obstetrics Gynecology 132 (3), e103–e119. 10.1097/aog.0000000000002833 30134425

[B2] Adam M. P. Polifka J. E. Friedman J. M. (2011). Evolving knowledge of the teratogenicity of medications in human pregnancy. Am. J. Med. Genetics Part C, Seminars Medical Genetics 157c (3), 175–182. 10.1002/ajmg.c.30313 21766440

[B3] Agarwal A. Aponte-Mellado A. Premkumar B. J. Shaman A. Gupta S. (2012). The effects of oxidative stress on female reproduction: a review. Reproduct. Biol. Endocrinol. 10, 10–49. 10.1186/1477-7827-10-49 22748101 PMC3527168

[B4] Bérard A. Sheehy O. Zhao J. P. Gorgui J. Bernatsky S. de Moura C. S. (2019). Associations between low- and high-dose oral fluconazole and pregnancy outcomes: 3 nested case-control studies. Can. Med. Assoc. J. 191 (7), E179–E187. 10.1503/cmaj.180963 30782643 PMC6379167

[B5] Chambers C. D. Krishnan J. A. Alba L. Albano J. D. Bryant A. S. Carver M. (2021). The safety of asthma medications during pregnancy and lactation: clinical management and research priorities. J. Allergy Clin Immunol 147 (6), 2009–2020. 10.1016/j.jaci.2021.02.037 33713765 PMC8185876

[B6] Chen T. Guestrin C. (2016). XGBoost: a scalable tree boosting system. In proceedings of the 22nd ACM SIGKDD international conference on knowledge discovery and data mining (San Francisco, CA, USA), 785–794. 10.1145/2939672.2939785

[B7] Chen H. L. S. Lee S.-I. (2021). Explaining models by propagating Shapley values of local components. Explain AI Healthc. Med. 914, 261–270. 10.1007/978-3-030-53352-6_24

[B8] Chen Y. Guo J. J. Steinbuch M. Lin X. Buncher C. R. Patel N. C. (2008). Comparison of sensitivity and timing of early signal detection of four frequently used signal detection methods. Pharm. Med. 22 (6), 359–365. 10.1007/BF03256733

[B9] Cho H. Jo H. Jeong Y. D. Jang W. Park J. Yim Y. (2025). Antipsychotic use during pregnancy and outcomes in pregnant individuals and newborns. J. Affect. Dis. 373, 495–504. 10.1016/j.jad.2024.12.102 39755128

[B10] Costa B. Gouveia M. J. Vale N. (2024). Oxidative stress induced by antivirals: implications for adverse outcomes during pregnancy and in newborns. Antioxidants (Basel) 13 (12), 1518. 10.3390/antiox13121518 39765846 PMC11727424

[B11] Dai F. F. Hu M. Zhang Y. W. Zhu R. H. Chen L. P. Li Z. D. (2022). Tnf-Α/anti-Tnf-Α drugs and its effect on pregnancy outcomes. Expert Rev. Mol. Med. 24, e26. 10.1017/erm.2022.18 35687009 PMC9884758

[B12] Dimitriadis E. Menkhorst E. Saito S. Kutteh W. H. Brosens J. J. (2020). Recurrent pregnancy loss. Nat. Reviews Dis. Primers 6 (1), 98. 10.1038/s41572-020-00228-z 33303732

[B13] Dimitsaki S. Natsiavas P. Jaulent M. C. (2024). Causal deep learning for the detection of adverse drug reactions: drug-induced acute kidney injury as a case study. Stud. Health Technolo. Informat. 316, 803–807. 10.3233/shti240533 39176914

[B14] Evans S. J. Waller P. C. Davis S. (2001). Use of proportional reporting ratios (Prrs) for signal generation from spontaneous adverse drug reaction reports. Pharmacoepidemiol. Drug Safety 10 (6), 483–486. 10.1002/pds.677 11828828

[B15] Fietz A. K. Onken M. Padberg S. Schaefer C. Dathe K. (2024). Impact of maternal first trimester treatment regimen on the outcome of valproate exposed pregnancies: an observational embryotox cohort study. Sci. Rep. 14 (1), 674. 10.1038/s41598-023-50669-1 38182639 PMC10770162

[B16] Garmendia J. V. De Sanctis C. V. Hajdúch M. De Sanctis J. B. (2025). Exploring the immunological aspects and treatments of recurrent pregnancy loss and recurrent implantation failure. Int. J. Mol. Sci. 26 (3), 1295. 10.3390/ijms26031295 39941063 PMC11818386

[B17] Guo X. Yi H. Li T. C. Wang Y. Wang H. Chen X. (2021). Role of vascular endothelial growth factor (Vegf) in human embryo implantation: clinical implications. Biomolecules 11 (2), 253. 10.3390/biom11020253 33578823 PMC7916576

[B18] Hoeltzenbein M. Bartz I. Fietz A. K. Lohse L. Onken M. Dathe K. (2024). Antiepileptic treatment with levetiracetam during the first trimester and pregnancy outcome: an observational study. Epilepsia 65 (1), 26–36. 10.1111/epi.17800 37857460

[B19] Hoffman R. M. Brummel S. S. Britto P. Pilotto J. H. Masheto G. Aurpibul L. (2019). Adverse pregnancy outcomes among women who conceive on antiretroviral therapy. Clin. Infect. Dis. 68 (2), 273–279. 10.1093/cid/ciy471 29868833 PMC6321847

[B20] Huybrechts K. F. Bateman B. T. Pawar A. Bessette L. G. Mogun H. Levin R. (2020). Maternal and fetal outcomes following exposure to duloxetine in pregnancy: cohort study. BMJ Clin. Res. Ed 368, m237. 10.1136/bmj.m237 32075794 PMC7190016

[B21] Ikumi N. M. Malaba T. R. Pillay K. Cohen M. C. Madlala H. P. Matjila M. (2021). Differential impact of antiretroviral therapy initiated before or during pregnancy on placenta pathology in HIV-positive women. Aids 35 (5), 717–726. 10.1097/qad.0000000000002824 33724257 PMC8630811

[B22] Jiang Y. Zhang Y. Li Y. Che Y. (2025). Research hotspots and trends in unexplained recurrent spontaneous abortion (Ursa) from 2014 to 2024: a bibliometric analysis. Front. Medicine 12, 1554875. 10.3389/fmed.2025.1554875 40438372 PMC12116356

[B23] Komaki F. Komaki Y. Micic D. Ido A. Sakuraba A. (2017). Outcome of pregnancy and neonatal complications with anti-tumor necrosis factor-Α use in females with immune mediated diseases; a systematic review and meta-analysis. J. Autoimmu. 76, 38–52. 10.1016/j.jaut.2016.11.004 27913060

[B24] Lashen H. Fear K. Sturdee D. W. (2004). Obesity is associated with increased risk of first trimester and recurrent miscarriage: matched case-control study. Hum. Reproduct. Oxf. Engl. 19 (7), 1644–1646. 10.1093/humrep/deh277 15142995

[B25] Lavertu A. Vora B. Giacomini K. M. Altman R. Rensi S. (2021). A new era in pharmacovigilance: toward real-world data and digital monitoring. Clin. Pharmacol. Therapeut. 109 (5), 1197–1202. 10.1002/cpt.2172 33492663 PMC8058244

[B26] Lee C. Y. Chen Y. P. (2019). Machine learning on adverse drug reactions for pharmacovigilance. Drug Dis. Today 24 (7), 1332–1343. 10.1016/j.drudis.2019.03.003 30876845

[B27] Lindquist M. Ståhl M. Bate A. Edwards I. R. Meyboom R. H. (2000). A retrospective evaluation of a data mining approach to aid finding new adverse drug reaction signals in the who international database. Drug Safety 23 (6), 533–542. 10.2165/00002018-200023060-00004 11144660

[B28] Liu B. Zhao C. Yang Y. Li J. Wu Y. Liu C. (2025). Parental preconception bmi and risk of adverse birth outcomes in 8 million parent-child triads: a nationwide population-based cohort study in China. Lancet Diab. Endocrinol. 13, 754–764. 10.1016/s2213-8587(25)00127-5 40651488

[B29] Lundberg S. M. Erion G. Chen H. DeGrave A. Prutkin J. M. Nair B. (2020). From local explanations to global understanding with explainable AI for trees. Nat. Machine Intell. 2 (1), 56–67. 10.1038/s42256-019-0138-9 32607472 PMC7326367

[B30] Lupattelli A. Spigset O. Twigg M. J. Zagorodnikova K. Mårdby A. C. Moretti M. E. (2014). Medication use in pregnancy: a cross-sectional, multinational web-based study. BMJ Open 4 (2), e004365. 10.1136/bmjopen-2013-004365 24534260 PMC3927801

[B31] Lupattelli A. Picinardi M. Cantarutti A. Nordeng H. (2020). Use and intentional avoidance of prescribed medications in pregnancy: a cross-sectional, web-based study among 926 women in Italy. Int. J. Environ. Res. Public Health 17 (11). 10.3390/ijerph17113830 32481641 PMC7312729

[B32] Magnus M. C. Wilcox A. J. Morken N. H. Weinberg C. R. Håberg S. E. (2019). Role of maternal age and pregnancy history in risk of miscarriage: prospective register based study. BMJ Clin. Res. Ed 364, l869. 10.1136/bmj.l869 30894356 PMC6425455

[B33] Mansour O. Russo R. G. Straub L. Bateman B. T. Gray K. J. Huybrechts K. F. (2024). Prescription medication use during pregnancy in the United States from 2011 to 2020: trends and safety evidence. Am. J. Obstetr. Gynecol. 231 (2), 250.e1–250.e16. 10.1016/j.ajog.2023.12.020 38128861 PMC11187710

[B34] Matsushita Y. Kuroda Y. Niwa S. Sonehara S. Hamada C. Yoshimura I. (2007). Criteria revision and performance comparison of three methods of signal detection applied to the spontaneous reporting database of a pharmaceutical manufacturer. Drug Safety 30 (8), 715–726. 10.2165/00002018-200730080-00008 17696584

[B35] Montastruc J. L. Sommet A. Bagheri H. Lapeyre-Mestre M. (2011). Benefits and strengths of the disproportionality analysis for identification of adverse drug reactions in a pharmacovigilance database. Br. J. Clin. Pharmacol. 72 (6), 905–908. 10.1111/j.1365-2125.2011.04037.x 21658092 PMC3244636

[B36] Mor G. Cardenas I. Abrahams V. Guller S. (2011). Inflammation and pregnancy: the role of the immune system at the implantation site. Ann. N. Y. Acad. Sci. 1221 (1), 80–87. 10.1111/j.1749-6632.2010.05938.x 21401634 PMC3078586

[B37] Muanda F. T. Sheehy O. Bérard A. (2017). Use of antibiotics during pregnancy and risk of spontaneous abortion. Can. Med. Assoc. J. 189 (17), E625–E633. 10.1503/cmaj.161020 28461374 PMC5415390

[B38] Nakane Y. Okuma T. Takahashi R. Sato Y. Wada T. Sato T. (1980). Multi-institutional study on the teratogenicity and fetal toxicity of antiepileptic drugs: a report of a Collaborative Study Group in Japan. Epilepsia 21 (6), 663–680. 10.1111/j.1528-1157.1980.tb04320.x 7439133

[B39] Newell M. L. Bunders M. J. (2013). Safety of antiretroviral drugs in pregnancy and breastfeeding for mother and child. Curr. Opinion HIV AIDS 8 (5), 504–510. 10.1097/COH.0b013e3283632b88 23743789

[B40] Nguyen J. Madonia V. Bland C. M. Stover K. R. Eiland L. S. Keating J. (2025). A review of antibiotic safety in pregnancy-2025 update. Pharmacotherapy 45 (4), 227–237. 10.1002/phar.70010 40105039 PMC11998890

[B41] Ornoy A. (2009). Valproic acid in pregnancy: how much are we endangering the embryo and fetus? Reprod. Toxicol. (Elmsford, NY) 28 (1), 1–10. 10.1016/j.reprotox.2009.02.014 19490988

[B42] Ornoy A. Echefu B. Becker M. (2023). Valproic acid in pregnancy revisited: neurobehavioral, biochemical and molecular changes affecting the embryo and fetus in humans and in animals: a narrative review. Int. J. Mol. Sci. 25 (1), 390. 10.3390/ijms25010390 38203562 PMC10779436

[B43] Pilipiec P. Liwicki M. Bota A. (2022). Using machine learning for pharmacovigilance: a systematic review. Pharmaceutics 14 (2), 266. 10.3390/pharmaceutics14020266 35213998 PMC8924891

[B44] Quenby S. Gallos I. D. Dhillon-Smith R. K. Podesek M. Stephenson M. D. Fisher J. (2021). Miscarriage matters: the epidemiological, physical, psychological, and economic costs of early pregnancy loss. Lancet (London, Engl.) 397 (10285), 1658–1667. 10.1016/s0140-6736(21)00682-6 33915094

[B45] Roldan Munoz S. Lupattelli A. de Vries S. T. Mol P. G. M. Nordeng H. (2020). Differences in medication beliefs between pregnant women using medication, or not, for chronic diseases: a cross-sectional, multinational, web-based study. BMJ Open 10 (2), e034529. 10.1136/bmjopen-2019-034529 32029496 PMC7044950

[B46] Rothman K. J. Lanes S. Sacks S. T. (2004). The reporting odds ratio and its advantages over the proportional reporting ratio. Pharmacoepidemiol. Drug Safety 13 (8), 519–523. 10.1002/pds.1001 15317031

[B47] Sethi N. Narayanan V. Saaid R. Ahmad Adlan A. S. Ngoi S. T. Teh C. S. J. (2025). Prevalence, risk factors, and adverse outcomes of bacterial vaginosis among pregnant women: a systematic review. BMC Pregnancy Childbirth 25 (1), 40. 10.1186/s12884-025-07144-8 39833700 PMC11744995

[B48] Shehata H. A. Nelson-Piercy C. (2001). Drugs in pregnancy. Drugs to avoid. Best Pract. Res. Clin. Obstetr. Gynaecol. 15 (6), 971–986. 10.1053/beog.2001.0241 11800536

[B49] Stjerna S. Huber-Mollema Y. Tomson T. Perucca E. Battino D. Craig J. (2024). Cognitive outcomes after fetal exposure to carbamazepine, lamotrigine, valproate or levetiracetam monotherapy: data from the eurap neurocognitive extension protocol. Epilepsy Behav. 159, 110024. 10.1016/j.yebeh.2024.110024 39217754

[B50] Stricker B. H. Tijssen J. G. (1992). Serum sickness-like reactions to cefaclor. J. Clin. Epidemiol. 45 (10), 1177–1184. 10.1016/0895-4356(92)90158-j 1474414

[B51] Szarfman A. Machado S. G. O'Neill R. T. (2002). Use of screening algorithms and computer systems to efficiently signal higher-than-expected combinations of drugs and events in the US FDA’s spontaneous reports database. Drug Safety 25 (6), 381–392. 10.2165/00002018-200225060-00001 12071774

[B52] Theron G. Brummel S. Fairlie L. Pinilla M. McCarthy K. Owor M. (2021). Pregnancy outcomes of women conceiving on antiretroviral therapy (Art) compared to those commenced on art during pregnancy. Clin. Infect. Dis. 73 (2), e312–e320. 10.1093/cid/ciaa805 32564058 PMC8516506

[B53] Toledano J. M. Puche-Juarez M. Moreno-Fernandez J. Gonzalez-Palacios P. Rivas A. Ochoa J. J. (2024). Implications of prenatal exposure to endocrine-disrupting chemicals in offspring development: a narrative review. Nutrients 16 (11), 1556. 10.3390/nu16111556 38892490 PMC11173790

[B54] Wall K. M. Haddad L. B. Mehta C. C. Golub E. T. Rahangdale L. Dionne-Odom J. (2019). Miscarriage among women in the United States women’s interagency HIV study, 1994–2017. Am. J. Obstetr. Gynecol. 221 (4), 347.e1–347.e13. 10.1016/j.ajog.2019.05.034 31136732 PMC6878114

[B55] Wang Q. Ma J. Lan Y. (2025a). Long-term trends in the global burden of maternal abortion and miscarriage from 1990 to 2021: joinpoint regression and age-period-cohort analysis. BMC Public Health 25 (1), 1554. 10.1186/s12889-025-22716-1 40287625 PMC12032735

[B56] Wang B. Zhuang S. Lin S. Lin J. Zeng W. Du B. (2025b). Analysis of risk factors for immune checkpoint inhibitor-associated liver injury: a retrospective analysis based on clinical study and real-world data. Hepatol. Int. 19 (5), 1172–1186. 10.1007/s12072-025-10783-w 40019709

[B57] Wang R. Dai F. Deng Z. Tang L. Liu H. Xia L. (2025c). Itga3 participates in the pathogenesis of recurrent spontaneous abortion by downregulating Ulk1-Mediated autophagy to inhibiting trophoblast function. Am. J. Physiol. Cell Physiol. 328 (6), C1941–C1956. 10.1152/ajpcell.00563.2024 39437445

[B58] Wang E. Liu Y. Wang Y. Han X. Zhou Y. Zhang L. (2025d). Comparative safety of antipsychotic medications and mood stabilizers during pregnancy: a systematic review and network meta-analysis of congenital malformations and prenatal outcomes. CNS Drugs 39 (1), 1–22. 10.1007/s40263-024-01131-x 39528870 PMC11695384

[B59] Wei M. M. Wang L. Zhao B. W. Su X. R. You Z. H. Huang D. S. (2025). Integrating transformer and graph attention network for Circrna-Mirna interaction prediction. IEEE J. Biomed. Health Inf. 29 (8), 6105–6113. 10.1109/jbhi.2025.3561197 40232907

[B60] Wei M. Wang L. Su X. Zhao B. You Z. (2026). Multi-Hop graph structural modeling for cancer-related Circrna-Mirna interaction prediction. Pattern Recognit. 170, 112078. 10.1016/j.patcog.2025.112078

[B61] Werler M. M. Kerr S. M. Ailes E. C. Reefhuis J. Gilboa S. M. Browne M. L. (2023). Patterns of prescription medication use during the first trimester of pregnancy in the United States, 1997-2018. Clin. Pharmacol. Therapeut. 114 (4), 836–844. 10.1002/cpt.2981 37356083 PMC10949220

[B62] Xu S. Cobzaru R. Finkelstein S. N. Welsch R. E. Ng K. Middleton L. (2024). Foundational model aided automatic high-throughput drug screening using self-controlled cohort study. medRxiv. 2024.08.04.24311480. 10.1101/2024.08.04.24311480 39148849 PMC11326319

[B63] Yang X. Tian Y. Zheng L. Luu T. Kwak-Kim J. (2022). The update immune-regulatory role of Pro- and anti-inflammatory cytokines in recurrent pregnancy losses. Int. J. Mol. Sci. 24 (1), 132. 10.3390/ijms24010132 36613575 PMC9820098

[B64] Zash R. Holmes L. Diseko M. Jacobson D. L. Brummel S. Mayondi G. (2019). Neural-tube defects and antiretroviral treatment regimens in Botswana. N. Engl. J. Med. 381 (9), 827–840. 10.1056/NEJMoa1905230 31329379 PMC6995896

